# Supplementary material for: Single Residue Substitution at N-Terminal Affects Temperature Stability and Activity of L2 Lipase
Source: Molecules. 2020 Jul 28;25(15):3433. doi: 10.3390/molecules25153433 (PMC7435863; doi:10.3390/molecules25153433)
Supplement: Supplementary file 1 [file molecules-25-03433-s001.pdf]

molecules

Supplementary Material

Single Residue Substitution at *N*-terminal Affects Temperature Stability and Activity of L2 Lipase

Noramirah Bukhari [1], Adam Thean Chor Leow [1,2], Raja Noor Zaliha Raja Abd Rahman[1,3] and Fairolniza Mohd Shariff [1,3,*]

[1] Enzyme and Microbial Technology Research Centre, Faculty of Biotechnology and Biomolecular Sciences, Universiti Putra Malaysia, Serdang 43400, Selangor, Malaysia; noramirahbukhari@gmail.com (N.B.); adamleow@upm.edu.my (A.T.C.L.); rnzaliha@upm.edu.my (R.N.Z.R.A.R.)
[2] Department of Cell and Molecular Biology, Faculty of Biotechnology and Biomolecular Sciences, Universiti Putra Malaysia, Serdang 43400, Selangor, Malaysia
[3] Department of Microbiology, Faculty of Biotechnology and Biomolecular Sciences, Universiti Putra Malaysia, Serdang 43400, Selangor, Malaysia
* Correspondence: fairolniza@upm.edu.my; Tel.: +60-3-96798279

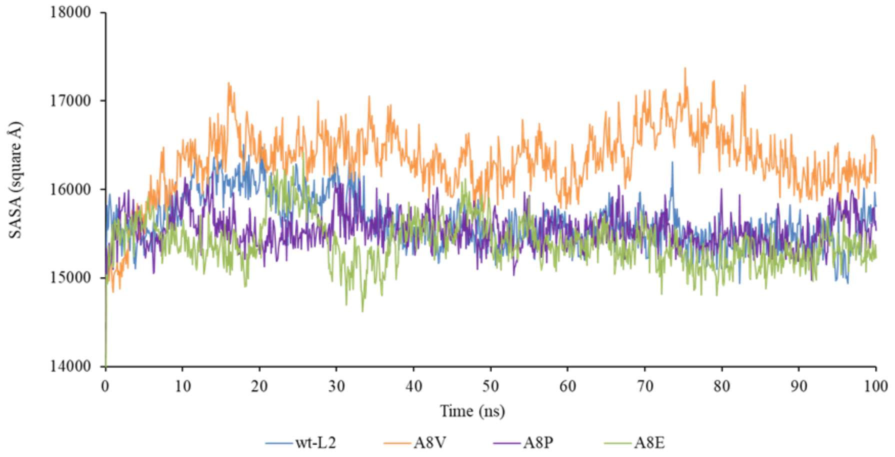

(a)

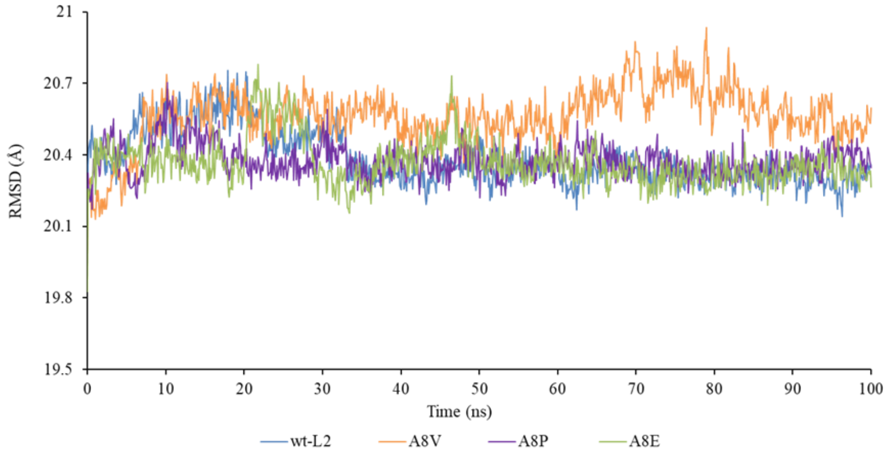

(b)

Figure S1. MD analysis of wt-L2 and mutant lipases for a period of 100 ns. (**a**) SASA analysis reflecting the surface of the global structure exposed to solvent and (**b**) Rgyration analysis indicating the compactness of the global structure.

Appendix B

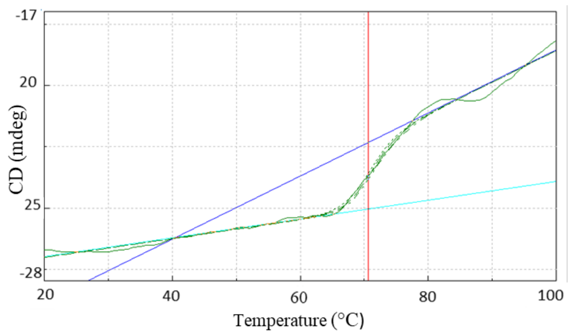

(a)

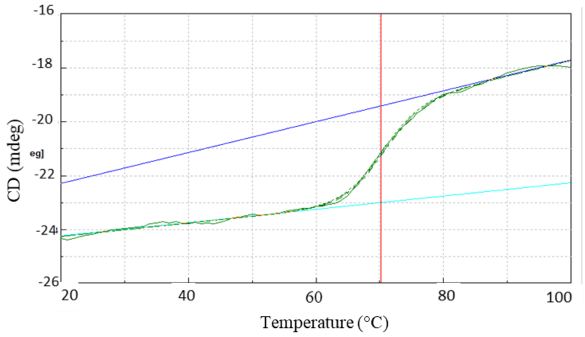

(b)

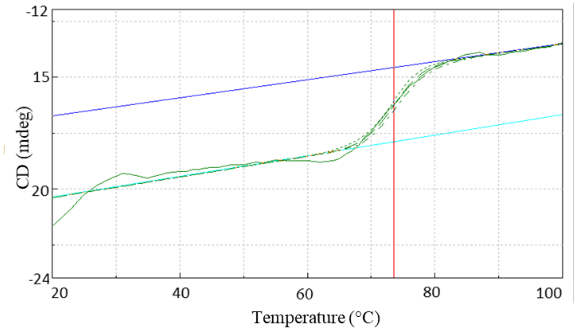

(c)

Figure S2. The thermal denaturation point (T_m) profile of (**a**) A8V, (**b**) A8P and (**c**) A8E. Denaturation analysis was executed at a temperature variable between 20 °C to 100 °C at the rate of 1 °C/min.

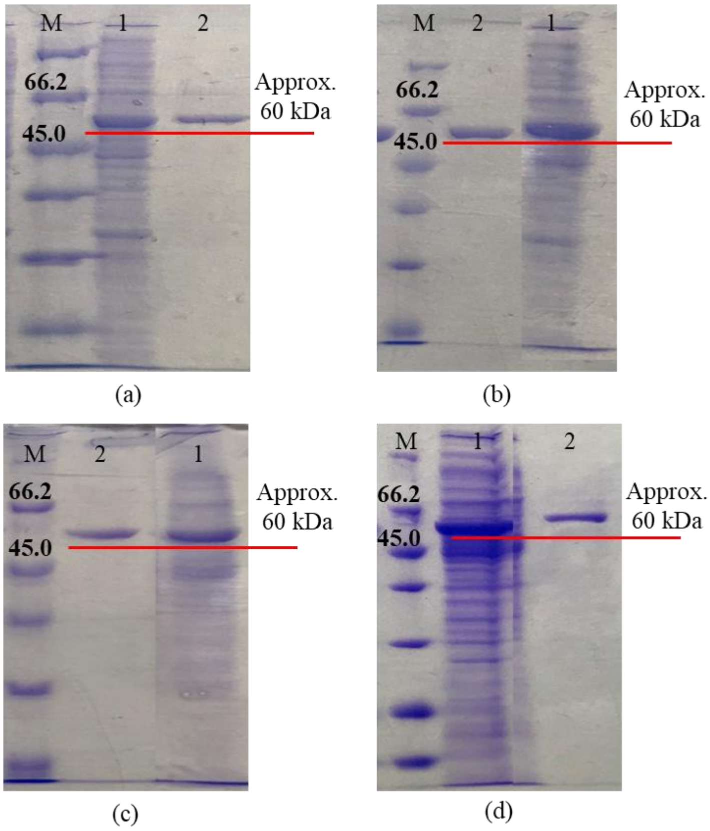

Figure S3. SDS-PAGE analysis of (a) wt-L2 and mutants (b) A8V, (c) A8P and (d) A8E showed expressed protein at size approximately 60 kDa. Lanes indicated by M is unstained protein ladder, 1 is the crude protein and 2 is purified lipase.

Table S1. The pKa of ionisable groups in the catalytic triad of the lipases, namely: Asp317 and His358.

Ionisable Group	wt-L2	A8V	A8P	A8E
Asp317	6.76	6.43	6.78	6.57
His358	−0.50	−0.35	−0.49	−0.46
